# Association between severity of obstructive sleep apnea and high‐sensitivity C‐reactive protein in patients with hypertrophic obstructive cardiomyopathy

**DOI:** 10.1002/clc.23385

**Published:** 2020-05-27

**Authors:** Juan Wang, Haobo Xu, Chao Guo, Xin Duan, Fenghuan Hu, Weixian Yang, Jingang Cui, Lei Song, Yushi Chun, Jiansong Yuan, Shubin Qiao

**Affiliations:** ^1^ Department of Cardiology, Fuwai Hospital, National Center for Cardiovascular Diseases Chinese Academy of Medical Sciences Beijing People's Republic of China

**Keywords:** high‐sensitivity C‐reactive protein, hypertrophic obstructive cardiomyopathy, inflammation, obstructive sleep apnea

## Abstract

**Background:**

Obstructive sleep apnea (OSA) is highly prevalent in patients with hypertrophic obstructive cardiomyopathy (HOCM). Inflammatory responses are increased in patients with OSA, meanwhile, inflammation is also associated with adverse outcomes in HOCM.

**Hypothesis:**

To investigate the association between severity of OSA and high‐sensitivity C‐reactive protein (hs‐CRP) in patients with HOCM.

**Methods:**

Three hundred and ninteen patients with HOCM who underwent sleep evaluations at Fuwai Hospital were retrospectively included between February 2010 and December 2018. Data from baseline clinical characteristics and polysomnography studies were collected.

**Results:**

OSA was present in 168 (52.7%). Patients with OSA were older, more likely to be male, had a higher body mass index and more clinical comorbidities. Patients with OSA had enlarged left ventricular diameter and similar left ventricular outflow tract obstruction compared with those without. In multivariate logistic analysis, apnea‐hypopnea index (OR, 1.024; 95% CI, 1.005‐1.044; *P* = .014), oxygen desaturation index (OR, 1.025; 95% CI, 1.004‐1.046; *P* = .018) and lowest oxygen saturation (OR, 0.951; 95% CI, 0.915‐0.989; *P* = .011) were independently associated with high risk hs‐CRP (>3 mg/L) after adjusting for confounders. In addition, decreasing lowest oxygen saturation (β = −.159, *P* = .004) was also independently correlated with increasing hs‐CRP concentrations in multivariate linear analysis after adjusting for confounders.

**Conclusions:**

Severity of OSA was independently associated with elevated hs‐CRP levels in patients with HOCM. Further studies are needed to evaluate the effects of treating OSA on hs‐CRP as well as clinical outcomes in these patients.

## 1 INTRODUCTION

Obstructive sleep apnea (OSA), characterized by oxygen desaturation and sleep fragmentation due to apneas and hypopneas during sleep, has been increasingly implicated in the pathogenesis and complications of cardiovascular disease.^1^, ^2^ Elevated systemic inflammation is seen in patients with OSA because of long‐term hypoxia, sympathetic activation, and oxidative stress.^3^, ^4^ In addition, treatment of OSA significantly reduces the inflammatory responses indicating an important role of inflammation in the pathophysiological processes of OSA.^5^, ^6^, ^7^

Hypertrophic cardiomyopathy (HCM) is one of the most common inherited cardiac diseases, characterized by ventricular hypertrophy, myofiber disarray, and fibrosis.^8^, ^9^, ^10^ Inflammation also plays a critical role in the development of myocardial remodeling as well as adverse outcomes in patients with HCM.^11^, ^12^ Furthermore, hypertrophic obstructive cardiomyopathy (HOCM), a phenotype of HCM with more symptoms and higher risk of sudden death, is associated with increased inflammatory responses compared with non‐obstructive HCM.^12^, ^13^ Considering that OSA is highly prevalent in patients with HCM ranging from 32% to 71%,^14^, ^15^ we propose that OSA be associated with elevated inflammation in HCM. Of the wide array of inflammatory biomarkers that have been studied, high‐sensitivity C‐reactive protein (hs‐CRP) has received the most attention for its use in screening and risk reclassification of cardiovascular disease.^16^ Therefore, the association of OSA with hs‐CRP in patients with HOCM, a more serious type of HCM, was investigated in this study.

## 2 MATERIALS AND METHODS

### 2.1 Study populations

This retrospective study included patients who were diagnosed with HOCM and underwent the first overnight diagnostic sleep examinations from in‐patient department at Fuwai Hospital between February 2010 and December 2018. The diagnosis of HOCM was made based on typical clinical, electrocardiographic, and echocardiographic features. Diagnostic criteria of HCM were consistent with the 2011 American Heart Association/American College of Cardiology and 2014 European Society of Cardiology guidelines, which mainly include unexplained septal hypertrophy with a thickness of 15 mm. We defined HOCM patients as who satisfied one of the following criteria based on echocardiography: (1) rest LVOT peak gradient ≥30 mmHg or (2) rest LVOT peak gradient <30 mmHg with provoked (valsalva maneuver, amyl nitrite or exercise) LVOT peak gradient ≥30 mmHg. Patients with both rest and provoked LVOT peak gradient <30 mmHg were defined as non‐obstructive HCM.

All patients were clinically stable who did not undergo changes in New York Heart Association (NYHA) functional class over the last 30 days and no patient was in NYHA class IV. Patients were further excluded if they had >50% central respiratory events, incomplete sleep recording data, were younger than 18 years old, or had previous septal reduction therapies (septal myectomy or alcohol septal ablation), or had history of heart transplantation surgery. No patient had systemic inflammatory disease, active infection, or trauma. All patients were not undergoing continuous positive airway pressure treatment before. Patient demographics and clinical data such as age, gender, body mass index, history of coronary artery disease, diabetes mellitus, hyperlipidemia, hypertension, and smoking were retrospectively reviewed.

All patients provided informed consent. The study was approved by the ethics committee of Fuwai Hospital. All studies were conducted in accordance with the ethical principles stated in the Declaration of Helsinki.

### 2.2 High‐sensitive C‐reactive protein assay

The baseline hs‐CRP values were collected from our medical records system. Briefly, fasting peripheral venous blood samples for hs‐CRP evaluation were obtained before polysomnography in the morning. After immediate centrifugation of the specimens, we stored them at 4°C prior to serum separation. The hs‐CRP was determined with an AUS5400 (Olympus, Japan) molecular analyzer at our clinical laboratory department. All samples were processed by technicians blinded to the samples. We used the recommended conventional cut‐off value for hs‐CRP of 3.0 mg/L provided by consensus conference of the American Heart Association (AHA) on the use of hs‐CRP in clinical practice.^17^ The hs‐CRP concentrations were categorized into two groups as follows: high risk level (>3 mg/L) and low risk level (≤3 mg/L).

### 2.3 Sleep study

An overnight polysomnography was performed in all the study populations using the portable monitoring system Embletta (Medcare Flaga, Reykjavik, Iceland). This device continuously recorded finger pulse oximetry, nasal airflow by an airflow pressure transducer, thoracic and abdominal movement, body position, snoring, heart rate and ECG, and has been validated against full polysomnography.^18^ The sleep was monitored automatically 30 minutes after the subjects went to bed. Apnea was defined when cessation of airflow or airflow reduction to ≤10% of the baseline value lasted for 10 seconds or more. Hypopnea was defined as a 50% or discernible decrement in airflow lasting 10 seconds with oxygen desaturation of 4%. Obstructive apneas were defined on basis of the presence of thoracic efforts. Apnea‐hypopnea index (AHI) was defined as the total number of apneas and hypopneas occurring per hour of sleep. Oxygen desaturation index (ODI) was defined as the number of oxygen level drops 4% from baseline per hour. Mean and minimal oxygen saturation (SaO2), average pulse frequency, and snoring proportion were also recorded. Diagnosis of OSA was made when the AHI in the recorded study was 5 events/hour or more, irrespectively to daytime OSA symptoms, which allowed objective evaluation of the disease severity.^19^, ^20^ Patients were classified into mild OSA (AHI: 5.0‐14.9 events/hour) and moderate to severe OSA (AHI ≥15.0 events/hour) based on widely accepted OSA severity threshold. OSA severity measures included AHI, ODI, longest apnea/hypopnea time, lowest SaO2, mean SaO2 and percent of total sleep time (TST) with SaO2 <90%.

### 2.4 Echocardiographic study

Echocardiography was performed using a GE Vivid 7 (GE Healthcare, Horten, Norway) with a multifrequency phased‐array transducer. Echocardiographic examinations were performed by one experienced physician. Diameters of the cardiac chambers were expressed as the maximum value of the anteroposterior diameter in cardiac cycles. The measurements of left ventricular volume, left ventricular ejection fraction, and left atrial diameter (LAD) were determined following the American Society of Echocardiography recommendations.^21^ The thickness of the interventricular septum and ventricular wall was determined during diastole. Representative thickness of the interventricular septum, which was usually the thickness of the point 25 mm under the right coronary sinus nadir, was recorded to indicate overall thickness. LVOT gradient was measured in the apical views by continuous‐wave Doppler echocardiography under resting conditions and during provocative maneuvers as previously reported.^22^

### 2.5 Statistical analysis

The results are expressed as mean ± SD, median (interquartile range), or number (percentage). Continuous variables were tested for normal distribution with the Kolmogorov‐Smirnov test. Comparison of categorical variables was performed using the χ^2^ or Fisher exact test, as appropriate. Differences among three groups were compared using one‐way analysis of variance or the Kruskal‐Wallis H test, as appropriate. Univariate and multivariate logistic regression analyses were used to determine the association between OSA severity measures and high risk hs‐CRP level. Significant variables in univariate analysis including age, body mass index (BMI), hypertension, fasting glucose, left ventricular end‐diastolic dimension (LVEDD), interventricular septum thickness (IVST), and supine sleep time, were included into multivariate regression analysis. Correlation between hs‐CRP concentrations and baseline characteristics were assessed through the use of the Pearson correlation coefficient for continuous variables and Spearman correlation coefficient for categorical variables. Multivariate linear regression analysis was used to identify relationship between OSA severity measures and hs‐CRP concentrations by adjusting for significant variables from correlation analysis such as age, BMI, hypertension, hyperlipidemia, LVEDD, fasting glucose, and supine sleep time. All reported probability values were 2‐tailed, and a *P* value of <.05 was considered statistically significant. SPSS version 24.0 (IBM Corp., Armonk, NY) and GraphPad Prism version 7.0 (GraphPad Software Inc., La Jolla, CA) were used for calculations and illustrations, respectively.

## 3 RESULTS

### 3.1 Population characteristics

A total of 319 patients with HOCM were included in the analysis (Supporting Information, Figure S1). One hundred and sixty‐eight (52.7%) were diagnosed with OSA and the median AHI value of the whole study population was markedly elevated (5.3, interquartile range [IQR] 1.5‐14.9 events/hour). Table 1 showed the demographic data, echocardiographic results, and sleep parameters of the study population grouped according to the severity of OSA. Those with more severe OSA were older, more likely to be male, had a higher BMI, be smokers, had higher NYHA cardiac function level, and had more clinical comorbidities such as hypertension, hyperlipidemia, coronary heart disease, stroke, and atrial fibrillation. The LVEDD was significantly enlarged and IVST decreased in patients with more severe OSA. There was no significant difference in LVOT gradient, LAD, and left ventricular ejection fraction among the study patients. The value of AHI, ODI, longest apnea/hypopnea time and percent of TST <90% were significantly increased, and level of lowest SaO2 and mean SaO2 were decreased with OSA severity.

**TABLE 1:** Clinical characteristics of patients with HOCM grouped according to OSA severity

Variables	None OSA (n = 151)	Mild OSA (n = 89)	Moderate to severe OSA (n = 79)	*P*‐value
Male	95 (62.9)	54 (60.7)	65 (82.3)	.004
Age (y)	44.8 ± 14.0	55.3 ± 10.5	51.8 ± 14.0	<.001
BMI (kg/m^2^)	24.6 ± 3.2	26.5 ± 3.1	27.5 ± 3.6	<.001
Cigarette use	53 (35.1)	37 (41.6)	44 (55.7)	.011
Hypertension	31 (20.5)	43 (48.3)	51 (64.6)	<.001
Hyperlipidemia	20 (13.2)	29 (32.6)	35 (44.3)	<.001
Diabetes	7 (4.6)	7 (7.9)	8 (10.1)	.270
Coronary heart disease	12 (7.9)	9 (10.1)	16 (20.3)	.019
Stroke	0 (0.0)	6 (6.7)	3 (3.8)	.008
NYHA class II‐III	105 (69.5)	74 (83.1)	63 (79.7)	.038
Familiar history of HCM	20 (10.1)	8 (9.0)	7 (8.9)	.468
Familiar history of SCD	5 (3.3)	3 (3.4)	5 (6.3)	.505
Syncope	18 (11.9)	18 (20.2)	13 (16.5)	.216
Atrial fibrillation	12 (7.9)	17 (19.1)	16 (20.3)	.011
Ventricular tachycardia	19 (12.6)	11 (12.4)	10 (12.7)	.998
High hs‐CRP level (%)	17 (11.3)	15 (16.9)	21 (26.6)	.012
Hs‐CRP (mg/L)	0.7 (0.3–1.3)	1.0 (0.5–2.1)	1.4 (0.6–3.1)	<.001
Fasting blood sugar (mmol/L)	4.6 ± 0.8	5.1 ± 1.2	5.1 ± 1.3	<.001
Total cholesterol (mmol/L)	4.4 ± 0.9	4.5 ± 1.0	4.4 ± 1.0	.466
Creatinine (mmol/L)	81.6 ± 15.1	84.3 ± 16.5	84.5 ± 16.9	.296
Echocardiographic data				
LVOTG at rest (mm Hg)	62.0 (41.5‐91.0)	61.0 (42.0‐87.0)	58.0 (34.0‐81.5)	.671
LAD (mm)	43.0 ± 6.1	43.8 ± 6.3	43.7 ± 6.8	.508
LVEDD (mm)	41.9 ± 4.3	43.8 ± 4.6	45.6 ± 4.8	<.001
IVST (mm)	19.9 ± 4.8	18.5 ± 5.1	18.2 ± 4.0	.011
LVEF (%)	68.7 ± 6.0	67.6 ± 5.5	68.8 ± 5.6	.283
PSG parameters				
AHI (events/h)	1.4 (0.6‐2.8)	8.3 (6.4‐11.4)	25.8 (19.9‐37.6)	<.001
ODI (events/h)	2.3 (1.0‐4.4)	8.4 (5.9‐11.3)	23.5 (18.4‐36.3)	<.001
Longest apnea/hypopnea time (s)	33.5 (24.0‐51.8)	62.5 (46.2‐79.5)	79.3 (61.2‐97.6)	<.001
Lowest SaO2 (%)	89.0 (86.0‐90.0)	85.0 (82.0‐88.0)	79.0 (74.0‐83.0)	<.001
Mean SaO2 (%)	94.4 (94.0‐95.0)	93.3 (92.9‐94.3)	93.0 (91.8‐94.0)	<.001
TST with SaO2 < 90% (%)	0.1 (0.0‐1.6)	1.8 (0.2‐8.3)	8.6 (2.9‐15.5)	<.001
Snoring time ratio (%)	2.8 (0.4‐6.7)	6.0 (1.5‐11.8)	12.9 (4.7‐19.2)	<.001
HR during sleep	71.6 ± 9.3	69.9 ± 10.7	72.2 ± 10.0	.281
Supine time (min)	208.0 (189.5‐305.0)	208.0 (155.0‐259.0)	208.0 (128.0‐252.5)	.039
Total recording time (min)	506.0 (467.0‐560.0)	491.0 (448.0‐533.0)	457.0 (410.5‐522.0)	.069

*Note:* Values are presented as mean ± SD, as median (interquartile range), or as n (%).

Abbreviations: AHI, apnea hypopnea index; BMI, body mass index; HCM, hypertrophic cardiomyopathy; hs‐CRP, high‐sensitivity C‐reactive protein; HR, heart rate; IVST, Interventricular septum thickness; LVOTG, left ventricular outflow tract gradient; LAD, left atrial diameter; LVEDD, left ventricular end‐diastolic dimension; LVEF, left ventricular ejection fraction; NYHA, New York Heart Association; ODI, oxygen desaturation index; OSA, obstructive sleep apnea; PSG, polysomnography; SCD, sudden cardiac death; SaO_2_, oxygen saturation; TST, total sleep time.

### 3.2 Association of OSA severity measures with high risk hs‐CRP level

**FIGURE 1:**
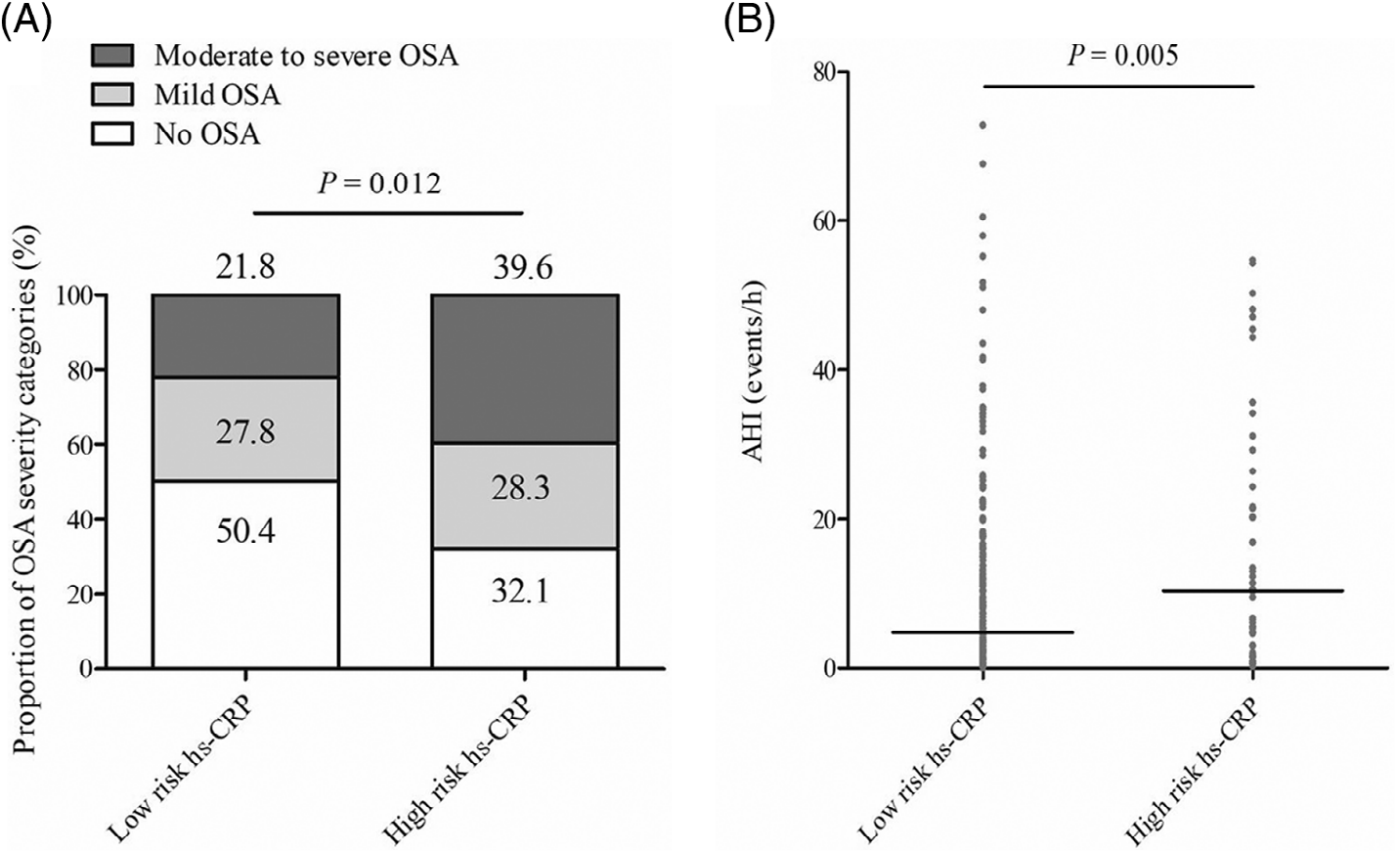
A, Prevalence of OSA in patients with low hs‐CRP level and high hs‐CRP level. B, Apnea‐hypopnea index in patients with low hs‐CRP level and high hs‐CRP level. Hs‐CRP, high‐sensitivity C‐reactive protein; OSA, obstructive sleep apnea

We then investigated the association between severity of OSA and high risk hs‐CRP level defined as a concentration >3 mg/L. Prevalence of patients with high risk hs‐CRP level (>3 mg/L) was significantly increased with OSA severity (*P* = .012, Table 1). Plasma hs‐CRP concentrations were also significantly higher in patients with moderate to severe OSA (median 1.4, IQR 0.6‐3.1 mg/L) than patients with no OSA (median 0.7, IQR 0.3‐1.3 mg/L) or with mild OSA (median 1.0, IQR 0.5‐2.1, *P* < .001, Table 1). Compared with patients in low risk hs‐CRP group, proportion of more severe OSA types as well as AHI values were significantly higher in high risk hs‐CRP group (*P* = .012 and *P* = .005, respectively, Figure 1). In multivariate analysis, after adjusting for age, BMI, hypertension, fasting glucose, LVEDD, IVST, and supine sleep time, AHI (odds ratio [OR], 1.024; 95% confidence interval [CI], 1.005‐1.044; *P* = .014), ODI (OR, 1.025; 95% CI, 1.004‐1.046; *P* = .018), and lowest SaO2 (OR, 0.951; 95% CI, 0.915‐0.989; *P* = .011) were independently associated with high risk hs‐CRP level, respectively (Table 2).

**TABLE 2:** Univariate and multivariate logistic regression analyses to identify the association between different OSA severity measures and patients with high hs‐CRP level

Variables	OR	95%CI	*P*‐value
Univariate			
Male	1.422	0.744‐2.612	.257
Age (y)	1.028	1.004‐1.053	.023
BMI (kg/m^2^)	1.111	1.022‐1.208	.014
Cigarette use	1.543	0.854‐2.788	.151
Hypertension	2.348	1.291‐4.271	.005
Hyperlipidemia	1.728	0.923‐3.236	.087
Diabetes	2.546	0.984‐6.588	.054
Coronary heart disease	2.059	0.930‐4.558	.075
Stroke	1.451	0.293‐7.186	.648
NYHA class II‐III	0.688	0.358‐1.321	.261
Familiar history of HCM	0.619	0.209‐1.833	.386
Familiar history of SCD	0.909	0.196‐4.225	.903
Syncope	0.976	0.429‐2.220	.953
Atrial fibrillation	1.100	0.481‐2.519	.821
Ventricular tachycardia	1.074	0.448‐2.577	.872
Fasting blood sugar (mmol/L)	1.353	1.068‐1.714	.012
Total cholesterol (mmol/L)	1.073	0.793‐1.452	.646
Creatinine (mmol/L)	1.006	0.988‐1.024	.528
LVOTG at rest (mm Hg)	0.996	0.987‐1.005	.390
LAD (mm)	0.990	0.944‐1.037	.664
LVEDD (mm)	1.102	1.033‐1.177	.003
IVST (mm)	0.932	0.870‐0.999	.048
LVEF (%)	0.951	0.904‐1.000	.050
AHI (events/h)	1.028	1.009‐1.047	.004
ODI (events/h)	1.029	1.009‐1.049	.005
Longest apnea/hypopnea time (s)	1.010	1.000‐1.019	.048
Lowest SaO2 (%)	0.951	0.917‐0.986	.007
Mean SaO2 (%)	0.949	0.806‐1.117	.530
TST with SaO2 < 90% (%)	1.017	0.994‐1.040	.145
Snoring time ratio (%)	1.021	0.997‐1.046	.094
HR during sleep (bpm)	1.025	0.982‐1.070	.254
Supine time (min)	0.995	0.992‐0.998	.002
Total recording time (min)	0.997	0.994‐1.000	.071
Multivariate			
AHI (events/h)	1.024	1.005–1.044	.014
ODI (events/h)	1.025	1.004–1.046	.018
Longest apnea/hypopnea time (s)	—	—	.202
Lowest SaO2 (%)	0.951	0.915–0.989	.011
Mean SaO2 (%)	—	—	.909
TST with SaO2 < 90% (%)	—	—	.291

*Note:* Data are presented as odds ratio (95% confidence interval). Significant variables from univariate analysis including age, BMI, hypertension, fasting blood sugar, LVEDD, IVST, and supine time, were adjusted in multivariate analysis for different OSA severity measures, respectively.

Abbreviations: AHI, apnea hypopnea index; BMI, body mass index; CI, confidence interval; Hs‐CRP, high‐sensitivity C‐reactive protein; HCM, hypertrophic cardiomyopathy; HR, heart rate; IVST, interventricular septum thickness; LVOTG, left ventricular outflow tract gradient; LAD, left atrial diameter; LVEDD, left ventricular end‐diastolic dimension; LVEF, left ventricular ejection fraction; NYHA, New York Heart Association; ODI, oxygen desaturation index; OSA, obstructive sleep apnea; OR, odds ratio; SCD, sudden cardiac death; SaO2, oxygen saturation; TST, total sleep time.

### 3.3 Linear regression analysis between OSA severity measures and hs‐CRP concentrations

**FIGURE 2:**
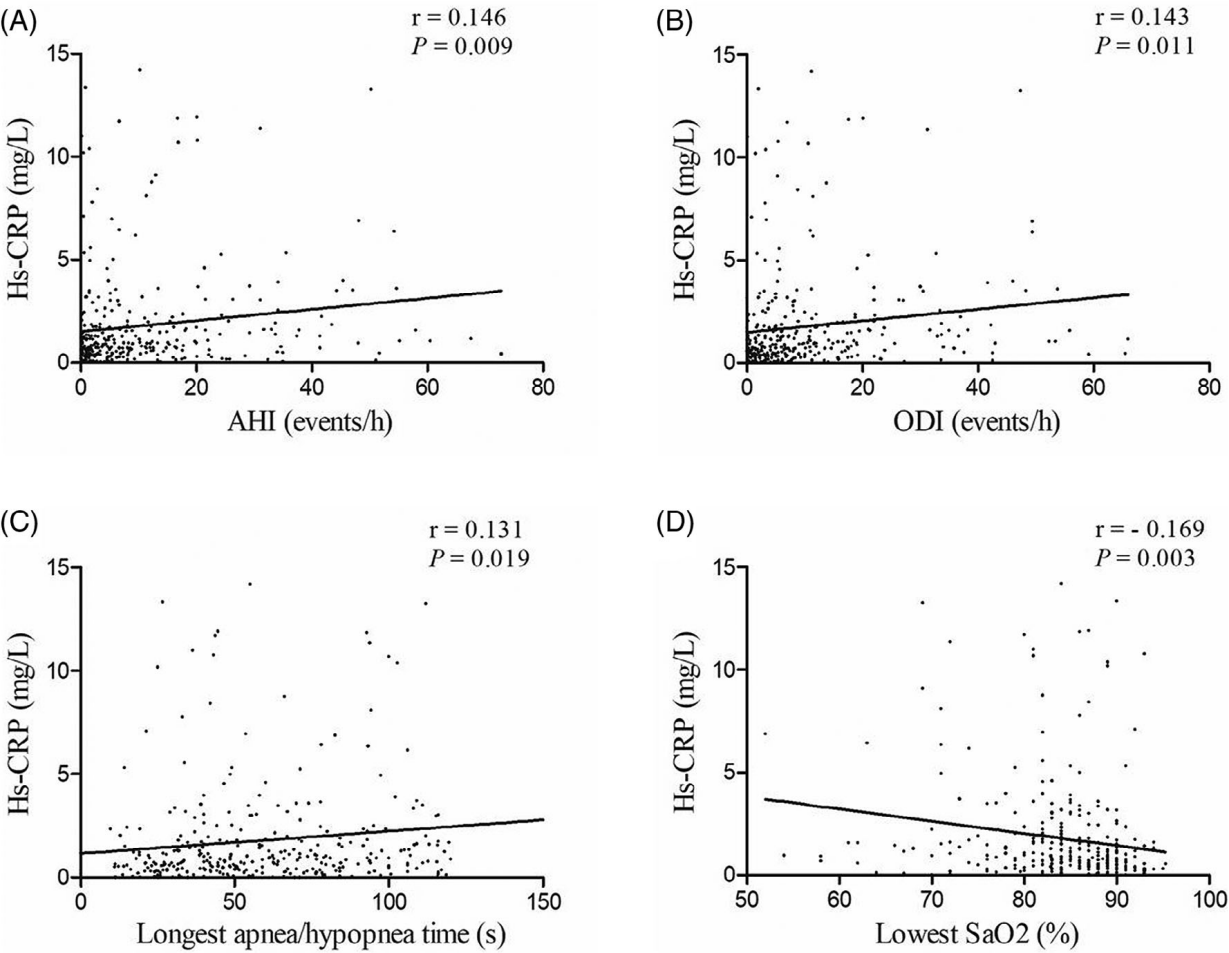
Correlation between serum levels of hs‐CRP and different severity measures of OSA. A, Correlation between hs‐CRP and AHI. B, Correlation between hs‐CRP and ODI. C, Correlation between hs‐CRP and Longest apnea/hyponea time. D, Correlation between hs‐CRP and lowest SaO2. AHI, apnea‐hypopnea index; Hs‐CRP, high‐sensitivity C‐reactive protein; OSA, obstructive sleep apnea; ODI, oxygen desaturation index; SaO2, oxygen saturation

Correlation between clinical characteristics and hs‐CRP concentrations were shown in Table S1. The value of hs‐CRP showed significant correlations with age (r = 0.134, *P* = .017), BMI (r = 0.170, *P* = .002), hypertension (r = 0.279, *P* < .001), hyperlipidemia (r = 0.203, *P* < .001), left ventricular end‐diastolic dimension (r = 0.144, *P* = .010), fasting glucose (r = 0.139, *P* = .013), and supine sleep time (r = −0.178, *P* = .001). Apnea‐hypopnea index, ODI, and longest apnea/hypopnea time were positively associated with increasing concentration of hs‐CRP, while lowest SaO2 showed inverse associations with hs‐CRP (Figure 2). In the multivariate linear analysis, decreasing lowest SaO2 (β = −.159, *P* = .004) was independently associated with increasing hs‐CRP concentrations after adjusting for significant variables in correlation analysis such as age, BMI, hypertension, hyperlipidemia, LVEDD, fasting glucose, and supine sleep time (Table 3).

**TABLE 3:** Multivariate linear regression analysis between different OSA severity measures and hs‐CRP value adjusting for significant variables from correlation analysis

Variables	β regression coefficients	*P*‐value
AHI (events/h)	.084	.152
ODI	.076	.202
Longest apnea/hypopnea time (s)	.078	.181
Lowest SaO2 (%)	−.159	.004
Mean SaO2 (%)	.001	.984
TST with SaO2 < 90% (%)	.069	.220

*Note:* Significant variables from correlation analysis were adjusted in multivariate linear regression including age, BMI, hypertension, hyperlipidemia, LVEDD, fasting blood sugar, and supine time.

Abbreviations: AHI, apnea hypopnea index; BMI, body mass index; Hs‐CRP, high‐sensitivity C‐reactive protein; LVEDD, left ventricular end‐diastolic dimension; OSA, obstructive sleep apnea; ODI, oxygen desaturation index; SaO_2_, oxygen saturation; TST, total sleep time.

## 4 DISCUSSION

This study showed that OSA was highly prevalent (52.7%) in this large HOCM population. The concentrations of hs‐CRP increased with severity of OSA. OSA severity measures such as AHI, ODI, and lowest SaO2, were independently associated with high risk hs‐CRP level (>3 mg/L) after adjusting for age, BMI, hypertension, fasting glucose, LVEDD, IVST, and supine sleep time. In addition, decreasing lowest SaO2 independently correlated with increasing hs‐CRP concentrations in multivariate linear analysis.

OSA is characterized by recurrent episodes of either partial or complete upper airway obstruction during sleep, leading to episodes of interruption of respiration and intermittent hypoxia.^23^ OSA is widely accepted as a risk factor for cardiovascular diseases such as hypertension, atrial fibrillation, ventricular arrhythmias, stroke, sudden cardiac death, and all‐cause mortality.^1^, ^2^ Until recently, studies showed that OSA was highly prevalent, ranging from 32% to 71%, in patients with HCM which was the most common genetic heart disease, occurring in one in 500 (0.2%) people.^14^, ^24^, ^25^, ^26^ Similarly, more than half of patients (52.7%) were diagnosed to have OSA in our study demonstrating that OSA is much common in patients with HOCM. The reason why OSA is highly prevalent in patients with HOCM is still unknown. The values of mean BMI in patients with mild OSA and moderate to severe OSA were 26.5 and 27.5 kg/m^2^, respectively, which indicated that obesity did not play a predominant role in propensity to OSA. It has been reported that overnight rostral fluid shift to the neck could contribute to upper airway obstruction.^27^ It also has been demonstrated that even in non‐obese healthy subjects, the shift of fluid into the nuchal structures may contribute to increase neck circumference and upper airway resistance.^28^ Rostral fluid shift is notable in patients with HOCM because the obstruction in left ventricular outflow tract increases left atrial and pulmonary pressure, resulting in increased blood volume and pressure in venous system,^29^ which aggravate this process.^14^ Therefore, overnight fluid shift could, at least in theory, play a role in the genesis of OSA among patients with HOCM. OSA was also reported to be associated with heart remodeling, atrial fibrillation, and ventricular tachycardia in HCM.^15^, ^25^ In this study, patients with HOCM and OSA were older, had a higher BMI and more clinical comorbidities which is consistent with previous studies.

Systemic inflammatory response is activated in patients with OSA because of intermittent hypoxia and reoxygenation, contributing to the cumulative burden of oxidative stress, generation of reactive oxygen species, and triggering of inflammatory cytokines.^30^ C‐reactive protein, a ubiquitous protein that synthesized in the liver, is a robust biomarker of underlying systemic inflammation and is mainly regulated by inflammatory cytokines, particularly interleukin 6 (IL‐6).^31^ Both increased plasma IL‐6 and CRP concentrations have been noted during hypoxic conditions.^32^ CRP measured by a highly sensitive assay, namely hs‐CRP, has considerable chemical stability, requires no special precautions for sampling, and has a relatively long half‐life.^33^ Thus, hs‐CRP has emerged as a leading biomarker of inflammation for clinical application. A plenty of studies had found increased levels of hs‐CRP in patients with OSA.^3^, ^4^ Additionally, hs‐CRP has also been regarded as a traditional risk factor in cardiovascular diseases.^16^ Previous studies showed that hs‐CRP level >3 mg/L was independently associated with a 60% excess risk in incident cardiovascular diseases as compared with hs‐CRP level <1 mg/L.^34^ Therefore, patients with hs‐CRP level >3 mg/L was considered with high risk. In this study, prevalence of patients with high risk hs‐CRP was increased with the severity of OSA. OSA severity measures such as AHI, ODI, and lowest SaO2 were independently associated with high risk hs‐CRP level. To our knowledge, this is the first study to demonstrate the association of OSA with elevated hs‐CRP levels in patients with HOCM.

It is remarkable that OSA, obstruction in upper airway, and HOCM, obstruction in LVOT, share common harmful pathways to the cardiovascular system.^14^ Firstly, overstimulation of the sympathetic nervous system and subsequently elevated catecholamine levels were seen both in OSA and HOCM.^35^ Secondly, large negative intrathoracic pressures generated because of increased inspiratory efforts in OSA could result in increasing left ventricular filling pressures, decreasing cardiac output, and worsening LVOT obstruction.^36^ Finally, both OSA and HOCM were associated with heart remodeling, arrythmias, and sudden cardiac death.^37^, ^38^ In our study, patients with OSA had worse NYHA cardiac function class, higher prevalence of atrial fibrillation, and enlarged left ventricles indicating a high grade of cardiac remodeling. Previous studies showed that plasma value of hs‐CRP was higher in patients with HCM compared with general population.^11^ Histological studies also demonstrated the infiltration of chronic inflammatory cells in the myocardium as well as the association of low‐grade inflammatory responses with myocardial fibrosis in HCM.^11^, ^39^ As hs‐CRP levels further increased with severity of OSA, we were led to speculate that elevated hs‐CRP might be a possible mechanism responsible for OSA‐related cardiovascular complications in HOCM. Future clinical trials are required to determine whether treatment of OSA could resolve inflammatory responses and improve prognosis in patients with HOCM.

This study has several limitations. Firstly, this study is a cross‐sectional study. Although our results suggested an independent association between OSA severity and hs‐CRP, the retrospective nature of this study limited our ability to determine a causal relationship. Secondly, this study was only designed to examine the hs‐CRP levels but not to other inflammatory biomarkers such as IL‐6 and tumor necrosis factor α, which have also been identified as stronger predictors of clinical events in patients with cardiovascular diseases. Thirdly, this is a single‐center study and our center is a tertiary national cardiovascular disease hospital, patients admitted to our hospital might be more symptomatic. Fourth, the values of hs‐CRP were measured at one‐time and we did not have serial measurements of hs‐CRP in this study. Finally, it is not possible to ensure that all confounding variables were fully adjusted in multivariate analysis. These facts limit the generalizability of our findings.

In conclusion, OSA is highly prevalent in patients with HOCM and hs‐CRP level increases with the severity of OSA. OSA severity measures such as AHI, ODI and lowest SaO2, were independently associated with high hs‐CRP level, a risk factor for cardiac death in this population. These results suggest that OSA should be screened for patients with HOCM and further studies are needed to evaluate the effects of treating OSA on hs‐CRP level as well as clinical outcomes for these patients over the long term.

## CONFLICT OF INTEREST

The authors declare no potential conflict of interests.

## Supporting information


**Figure S1** Study flow diagram. HOCM, hypertrophic obstructive cardiomyopathy; PSG, polysomnography.

**Table S1** Correlation analysis between hs‐CRP and clinical variables.
