# Supplementary figures and images for: Association between severity of obstructive sleep apnea and high‐sensitivity C‐reactive protein in patients with hypertrophic obstructive cardiomyopathy
Source: Clin Cardiol. 2020 May 27;43(7):803–11. doi: 10.1002/clc.23385 (PMC7368348; doi:10.1002/clc.23385)

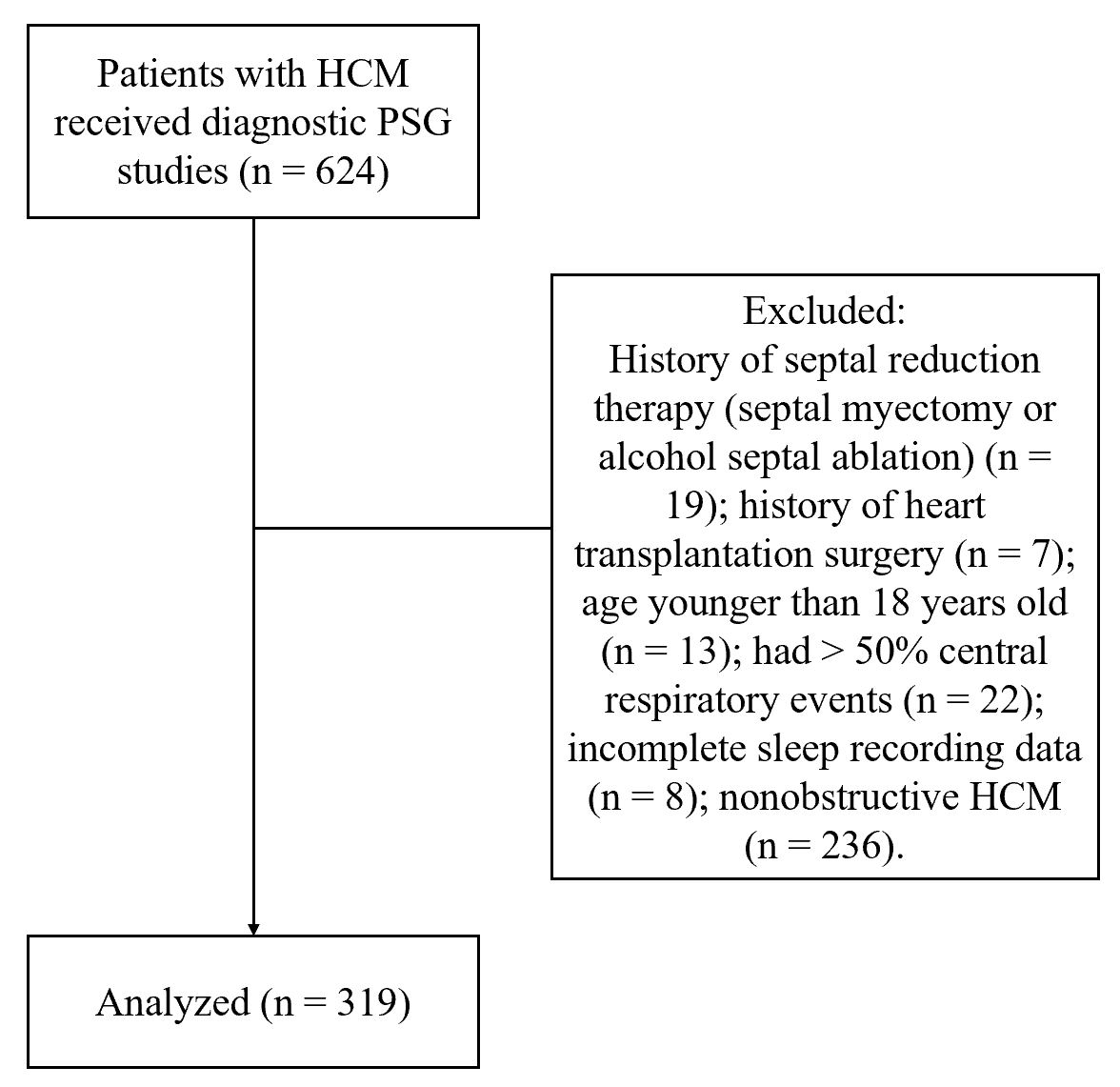

Supplement: Supplementary file 1 — Figure S1 Study flow diagram. HOCM, hypertrophic obstructive cardiomyopathy; PSG, polysomnography. [file CLC-43-803-s001.tif]
