# Supplementary material for: Association between severity of obstructive sleep apnea and high‐sensitivity C‐reactive protein in patients with hypertrophic obstructive cardiomyopathy
Source: Clin Cardiol. 2020 May 27;43(7):803–11. doi: 10.1002/clc.23385 (PMC7368348; doi:10.1002/clc.23385)
Supplement: Supplementary file 2 — Table S1 Correlation analysis between hs‐CRP and clinical variables. [file CLC-43-803-s002.docx]

| Table S1. Correlation analysis between hs-CRP and clinical variables. | | |
| --- | --- | --- |
| Variables | r | P-value |
| Male | 0.022 | 0.697 |
| Age (y) | 0.134 | 0.017 |
| BMI (kg/m2) | 0.170 | 0.002 |
| Cigarette use | 0.095 | 0.091 |
| Hypertension | 0.279 | ＜0.001 |
| Hyperlipidemia | 0.203 | ＜0.001 |
| Diabetes | 0.098 | 0.080 |
| Coronary heart disease | 0.098 | 0.080 |
| Stroke | 0.108 | 0.054 |
| NYHA class II - III | -0.030 | 0.593 |
| Familiar history of HCM | 0.022 | 0.698 |
| Familiar history of SCD | -0.043 | 0.441 |
| Syncope | -0.042 | 0.495 |
| Atrial fibrillation | 0.028 | 0.619 |
| Ventricular tachycardia | -0.054 | 0.338 |
| Fasting blood sugar (mmol/L) | 0.139 | 0.013 |
| Total cholesterol (mmol/L) | 0.052 | 0.357 |
| Creatinine (mmol/L) | 0.104 | 0.065 |
| LVOTG at rest (mm Hg) | -0.060 | 0.284 |
| LAD (mm) | 0.004 | 0.940 |
| LVEDD (mm) | 0.144 | 0.010 |
| IVST (mm) | -0.075 | 0.184 |
| LVEF (%) | -0.084 | 0.133 |
| AHI (events/h) | 0.146 | 0.009 |
| ODI (events/h) | 0.143 | 0.011 |
| Longest apnea/hypopnea time (s) | 0.131 | 0.019 |
| Lowest SaO2 (%) | -0.169 | 0.003 |
| Mean SaO2 (%) | -0.045 | 0.422 |
| TST with SaO2 < 90% (%) | 0.098 | 0.079 |
| Snoring time ratio (%) | 0.069 | 0.217 |
| HR during sleep | 0.034 | 0.548 |
| Supine time (min) | -0.178 | 0.001 |
| Total recording time (min) | -0.071 | 0.207 |

Hs-CRP: high-sensitivity C-reactive protein; BMI: body mass index; NYHA: New York Heart Association; HCM: hypertrophic cardiomyopathy; SCD: sudden cardiac death; LVOTG: left ventricular outflow tract gradient; LAD: left atrial diameter; LVEDD: left ventricular end-diastolic dimension; IVST: Interventricular septum thickness; LVEF: left ventricular ejection fraction; AHI: apnea hypopnea index; ODI: oxygen desaturation index; SaO_2_: oxygen saturation; TST: total sleep time; HR: heart rate.
